# 6-Gingerol ameliorates high-fat, high-sucrose diet-induced metabolic dysfunction and depressive-like behaviors by attenuating neuroinflammation and oxidative stress

**DOI:** 10.1007/s11011-025-01782-9

**Published:** 2026-02-03

**Authors:** Hend A. Essa, Abeer E. El-Metwally

**Affiliations:** 1 Nutrition and Food Sciences Department, Food Industries and Nutrition Research Institute, National Research Centre, 33 El Bohouth St, Dokki, Giza, 12622 Egypt; 2 Pathology Department, Animal Reproduction Research Institute (A.R.R.I.), Cairo, Egypt

**Keywords:** 6-Gingerol, Depression like behavior, High-fat/high-sucrose diet, Metabolic syndrome, Oxidative stress, Brain-Derived neurotrophic factor, Rats

## Abstract

High-calorie diets cause metabolic syndrome, obesity, and emotional disturbances, with neurological consequences. These prevalent conditions impair both peripheral and central nervous system function, elevating depression risk. These complications represent prevalent chronic conditions in modern society. The bioactive compound 6-gingerol demonstrates antioxidant and anti-inflammatory properties. This study investigated 6-gingerol’s protective effects against depression-like behavior and metabolic syndrome induced by a high-fat, high-sucrose diet (HFHS) in rats. Male Sprague-Dawley rats were randomly divided into six groups (*n* = 8/group): normal control (balanced diet; 4.12 kcal/g), HFHS-fed (5.57 kcal/g + 0.8 kcal/mL), low-dose (100 mg/kg/day, oral 6-gingerol), high-dose (200 mg/kg/day, oral 6-gingerol), and HFHS-fed groups receiving low-dose (100 mg/kg/day) or high-dose (200 mg/kg/day) oral 6-gingerol for ten weeks. Behavioral tests (forced swim, tail suspension) were conducted. Measured parameters included fasting blood glucose, serum lipids, insulin, leptin, hs-CRP, neurochemical markers (BDNF, GABA, AChE), neurotransmitters (serotonin, dopamine), oxidative stress markers, and inflammatory cytokines. Brain tissues were analyzed histopathologically and immunohistochemically. Both 6-gingerol doses significantly (*p* < 0.05) improved hyperglycemia, insulin resistance, dyslipidemia, hs-CRP, and leptin levels. Glutathione and glutathione peroxidase increased, while malondialdehyde, protein carbonyl, nitric oxide, TNF-α, and IL-6 significantly decreased. Immobility time in forced swim and tail suspension tests was significantly reduced. Neurochemical and neurotransmitter markers in brain tissue improved significantly (*p* < 0.05). GFAP and iNOS expression was reduced (*p* < 0.05), showing dose dependency. Histopathological and histochemical analyses confirmed 6-gingerol’s neuroprotection. 6-Gingerol, especially at high dose, effectively alleviated HFHS-induced metabolic syndrome, and depression-like behavior. These findings underscore its potential as a prophylactic against neurobehavioral deficits, and diet-associated depression.

**Graphical abstract:**

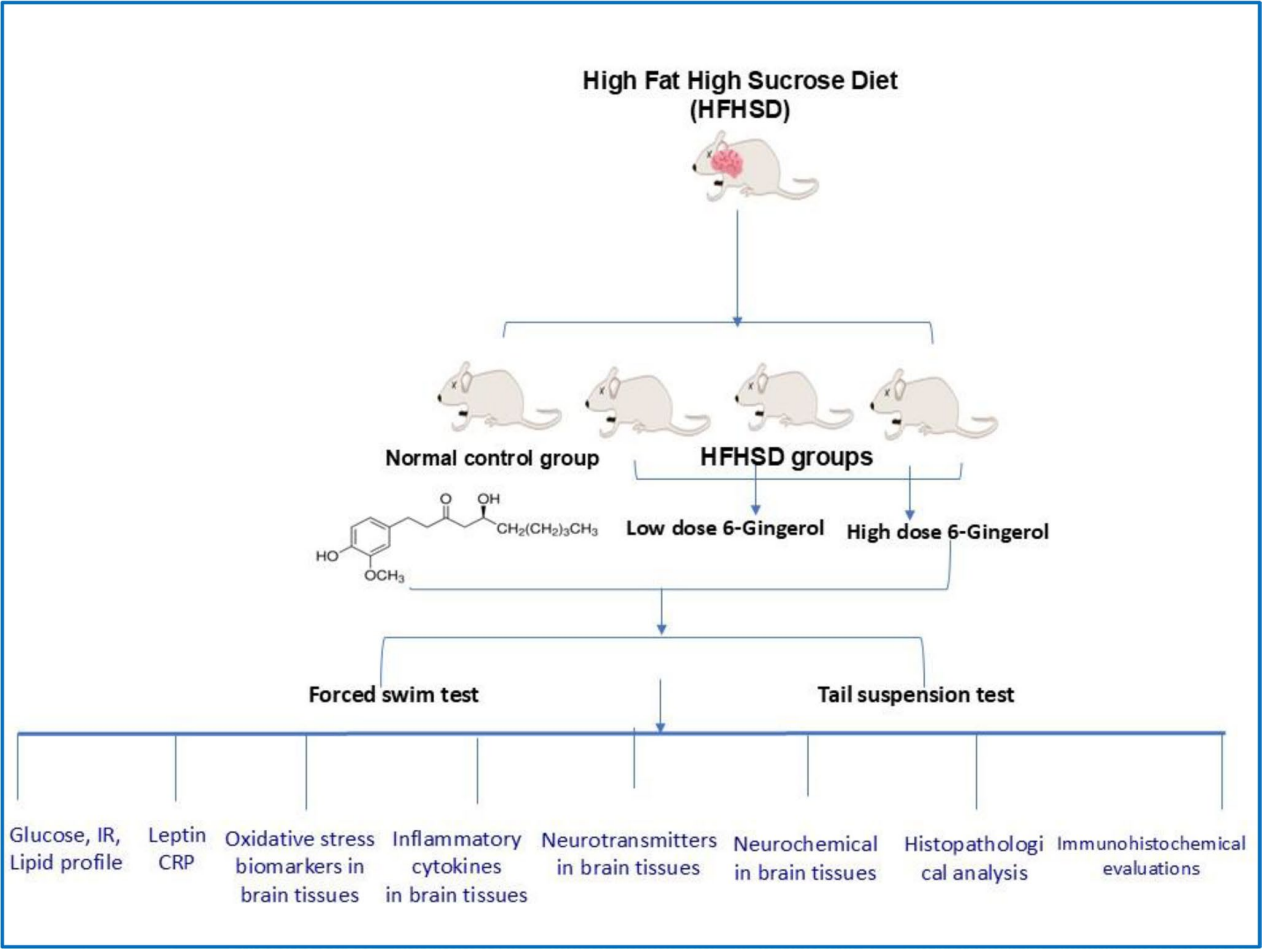

## Introduction

Dietary choices are critical determinants of overall health, with high-calorie diets, characterized by elevated fat and sugar content—commonly termed the Western Diet (WD)—being a well-established precursor to overweight and obesity (World Health Organization 2022). This dietary pattern, coupled with an imbalance between energy intake and expenditure, has reached pandemic levels, making it crucial to understand the full spectrum of resulting metabolic and neurological alterations (Ledreux et al. 2016). Prolonged exposure to obesogenic diets, rich in saturated fat and sucrose (HFHS), not only leads to metabolic syndrome (MetS) and type 2 diabetes mellitus (T2DM) but also frequently coexists with major depressive disorder (MDD) and anxiety (Shinkov et al. 2018).

Consumption of the WD is stronglpy implicated in promoting neuroinflammatory processes and oxidative stress in the brain (Skinner 2016). Neuroinflammation involves the activation of glial cells, such as astrocytes, which respond to metabolic insults through reactive astrogliosis—marked by the increase in the glial fibrillary acidic protein (GFAP) marker (Nguyen et al. 2014; Mohr et al. 2021). Prior investigations employing rodent models subjected to high fat high sucrose (HFHS) diets have shown that this diet-induced peripheral inflammation rapidly translates into central nervous system dysfunction (Mohr et al. 2021). The brain is particularly susceptible to oxidative damage due to its high oxygen demand, high mitochondrial content, and limited antioxidant defense system (Wojsiat et al. 2018).

Severe depression is linked to neurodegenerative processes driven by this chronic inflammation and oxidative stress, which interfere with synaptic function and neuroplasticity. A key component affected is Brain-Derived Neurotrophic Factor (BDNF), a critical neurotrophin that promotes neuronal growth, regulates synaptic plasticity, and is central to mood regulation and memory (Lu et al. 2014; Numakawa et al. 2018). The neurotrophic hypothesis of depression highlights the direct link between decreased BDNF levels and increased susceptibility to major depressive disorder (MDD), supported by observations of decreased hippocampal volume in depressed patients (Martinowich et al. 2007; Ledreux et al. 2016). Furthermore, neurotransmitter systems (such as Serotonin and Dopamine) are compromised by the same inflammatory and oxidative milieu, contributing directly to core depressive symptoms (Burtscher et al. 2022).

Given the intersection of metabolic dysfunction, oxidative stress, and neuroinflammation in depression, natural antioxidants with multiple health benefits, such as 6-gingerol (C17​H26​O4​), have garnered significant attention. 6-gingerol, chemically known as (S)−5-hydroxy-1-(4-hydroxy-3-methoxyphenol)−3-decanone, is an aromatic polyphenol and a key bioactive component of ginger (Zingiber officinale Roscoe), belonging to the family Zingiberaceae. It has been found to exhibit a range of interesting pharmacological effects, including antioxidant, anti-inflammatory, anti-obesity, antidiabetic, and cardioprotective properties (Gandhi et al. 2023; Esrefoglu and Bak 2014). Most recently, a study demonstrated that 6-gingerol ameliorates adiposity and obesity-associated inflammation in white adipose tissue, linked to the regulation of adipokines in diet-induced obese mice (Hong et al. 2023). Due to its diverse beneficial effects and low toxicity, 6-gingerol is considered a promising candidate (Sarrafan et al. 2021).

Given the role of Western diets—high in fat and sugar—and metabolic dysfunction in depression risk, there is an urgent need for preventive interventions. While 6-gingerol - a bioactive component of ginger - demonstrates metabolic benefits, its neuroprotective potential against diet-induced depression remains unexplored. This study evaluates the effects of low and high doses of 6-gingerol on depression-like behaviors (assessed using the forced swim test [FST] and tail suspension test [TST]), neurochemical markers (BDNF, GABA, AChE), neurotransmitter levels, oxidative/inflammatory status, and brain histopathology in a high-fat/high-sugar (HFHS) diet-induced rat model. The rodent system enables rigorous investigation of these multifactorial interactions through direct tissue analysis. Our findings may position 6-gingerol as a potential prophylactic dietary component to counteract neurological impairments linked to Western-style diets.

## Materials and methods

### Materials

#### Animals

Adult male Sprague Dawley rats aged 3–4 weeks (body weight 110–130 g) were procured from the Animal Health Research Institute, Egypt. The rats underwent a one-week acclimatization period in the institute’s animal facility under controlled environmental conditions. Animals were individually housed in stainless steel cages. Environmental parameters were maintained at 23 ± 2 °C ambient temperature with 55–60% relative humidity and a regulated 12-hour light/dark photoperiod. Throughout the study period, all animals had free access to laboratory diet and tap water.

#### Diet preparations

This study employed two primary dietary regimens. The balanced diet formulated according to AIN-93 nutritional standards (Reeves et al. 1993). The high-fat high-sucrose (HFHS) diet, rats receiving the HFHS diet were provided with unlimited access to a 20% sucrose aqueous solution (De Paula et al. 2024; Mota et al. 2023a, b). The HFHS diet composition delivered 56.55% of calories from fat (beef tallow) and 34.83% from carbohydrates, supplemented with 20% (w/v) sucrose water. Detailed formulations are presented in Table 1.

**Table 1:** Composition of balanced, and high-fat high-sucrose diets (g/100 g)

Ingredients	Balanced Diet	HFHS Diet
Casein	12	12
Corn oil	10	-
Beef tallow	-	35
Sucrose	10	10
Corn starch	58.5	38.5
AIN Mineral mix	3.5	3.5
AIN Vitamin mix	1	1
Cellulose	5	-
Additional supplement	-	20% (w/v) sucrose water
Total Calories (kCal/g)	4.12 kcal/g	**5.57** kcal/g + 0.8 Kcal/mL from sucrose water

#### Ethical approval

The study protocol received approval from the Institutional Animal Care and Use Committee of the Agriculture Research Centre (ethical approval number: ARC-ARRI-158-24). All experimental procedures strictly adhered to the National Institutes of Health guidelines for laboratory animal care and use (Garber et al. 2011). All experiments were conducted in accordance with relevant guidelines and regulations, including the ARRIVE guidelines, U.K. Animals Act 1986 and associated guideline, EU Directive.

### Methods

#### Experimental design

Following a one-week acclimatization period, 48 rats were randomly allocated into four experimental groups (*n* = 8 per group):

- **Group 1 (Normal control)**: Received balanced diet with daily oral administration of 1 mL/kg corn oil.
- **Group 2 (HFHS control)**: Fed high-fat high-sucrose (HFHS) diet with ad libitum access to 20% sucrose solution and daily 1mL/kg corn oil for 10 weeks.
- **Group 3 (6-Gingerol low dose)**: Maintained on the balanced diet and received 6-gingerol (C₁₇H₂₆O₄; G1045, molecular weight: 294.39; Sigma-Aldrich, St. Louis, MO, USA) at 100 mg/kg in corn oil (Aboismaiel et al. 2024; Gunawan et al. 2023).
- **Group 4 (6-Gingerol high dose)**: Maintained on the balanced diet and received 6-gingerol (C₁₇H₂₆O₄; G1045, molecular weight: 294.39; Sigma-Aldrich, St. Louis, MO, USA) at 200 mg/kg in corn oil (Aboismaiel et al. 2024; Gunawan et al. 2023).
- **Group 5 (6-Gingerol low dose)**: Maintained on the HFHS diet with 15% sucrose solution and received 6-gingerol (C₁₇H₂₆O₄; G1046, molecular weight: 294.39; Sigma-Aldrich, St. Louis, MO, USA) at 100 mg/kg in corn oil (Aboismaiel et al. 2024; Gunawan et al. 2023) by oral gavage once daily for 10 weeks.
- **Group 6 (6-Gingerol high dose)**: Received Maintained on the HFHS diet with 15% sucrose solution and received 6-gingerol (C₁₇H₂₆O₄; G1046, molecular weight: 294.39; Sigma-Aldrich, St. Louis, MO, USA) at 200 mg/kg in corn oil (Aboismaiel et al. 2024; Gunawan et al. 2023) by oral gavage once daily for 10 weeks.

All animals had continuous access to water and their respective diets. Food and water were replenished every day. Final assessments comprised: final body weight, total food intake, and body weight gain were measured. The Feed efficiency ratio (weight gain/total food intake). The relative weight of the brain was calculated as (brain weight/final body weight) × 100. (Chapman et al. 1959). The body mass index (BMI), was calculated using the formula: BMI = body weight (g)/[length (nose to anus, cm)]².

#### Assessment of depression-like behaviors

Behavioral testing commenced 24 h following the final treatment administration. All apparatuses were sanitized with 70% ethanol between trials to eliminate olfactory cues and allowed to dry for one minute. A trained observer, blinded to treatment conditions, conducted all behavioral assessments manually.

A ***Forced Swim Test (FST)***.
  The FST represents the gold standard for evaluating depressive-like behavior in rodents. After the 10-week intervention, animals were individually placed in transparent glass cylinders (20 cm diameter × 41 cm height) filled with water (25 ± 1 °C) to a depth of 30 cm, preventing contact with the bottom. Following a 15-minute acclimatization session, rats were dried, warmed, and returned to their home cages. After 24 h, a 5-minute test session was conducted under identical conditions with cleaned cylinders between subjects. Three behavioral parameters were quantified: Immobility time: Periods of passive floating with only minimal movements to maintain buoyancy. Swimming time: Active horizontal movements using forepaws. Climbing time: Vigorous vertical movements against cylinder walls using all limbs (Buddenberg et al. 2009; Detke et al. 1995).
B ***Tail Suspension Test (TST)***.
  In brief, rats were suspended approximately 28 ± 2 cm above the floor by securing their tails 2 cm from the tip. Throughout the 6-minute test period, immobility time was automatically recorded (Crowley et al. 2004; Shang et al. 2015). Immobility is characterized by the cessation of all active movements, including limb swinging (both forelimbs and hindlimbs) and body twisting, in the suspended animal.

#### Blood and brain sampling

Following behavioral assessments, animals underwent a 12-hour fasting period prior to terminal procedures, and the sucrose aqueous water was replaced with tap water. Anesthesia was induced via intramuscular ketamine hydrochloride (35 mg/kg). Blood samples were collected from the retro-orbital plexus of the anesthetized rats’ eyes. The sera were separated by centrifugation (3000 rpm, 15 min, 4 °C; Laborezentrifugen 2k15, Sigma, Germany) and stored at −20 °C. for subsequent biochemical analyses. Following blood collection, rats were euthanized via cervical dislocation.

Whole brains were rapidly excised and dissected into cerebrum and cerebellum regions. Each region was partitioned for: (a) Biochemical analysis, tissue homogenates (10% w/v in ice-cold phosphate buffer, pH 7.4), were prepared using a mechanical homogenizer (MPW-120, BitLab Medical Instruments, Poland), centrifuged (4000 rpm, 10 min, 4 °C) using a cooling centrifuge (Laboratory Centrifuge, 2K15, Sigma Co., Germany) (Essa et al. 2025). The supernatant was collected, stored at −20 °C for oxidative/antioxidant markers, neurotransmitter quantification, and cytokine profiling (b), histopathological examination: Tissue samples were fixed in 10% neutral buffered formalin.

#### Metabolic parameter analysis

Fasting blood glucose levels were quantified via enzymatic colorimetric assay (Trinder 1969). The lipid profile was assessed by measuring total cholesterol (TC) (Watson 1960), HDL-C (Burstein et al. 1970), LDL-C (Schriewer et al. 1984), and triglycerides (TG) (Megraw et al. 1979). VLDL-C was derived as TG/5, and non-HDL-C was calculated as TC minus HDL-C. The atherogenic index (TC/HDL-C ratio) was also determined. All analyses employed commercial kits from Spectrum Diagnostic (MDSS GmbH, Hannover, Germany; catalog numbers 250 001, 230 006, 266 001, 280 001, 314 002, respectively). Serum inflammatory and metabolic markers were evaluated using rat-specific enzyme-linked immunosorbent assay (ELISA) kits. These included hs-CRP (Catalog No. SL0348Ra), Leptin (Catalog No. SL0441Ra), and Insulin (Catalog No. SL0373Ra), all from Sunlong Biotechnology Co., LTD, Hangzhou, China. Insulin resistance was calculated using the following formula: HOMA-IR index = [fasting glucose (mg/dL) × fasting insulin (µU/mL)/405] (Matthews et al. 1985).

#### Assessment of oxidative/antioxidant parameters

Brain tissue oxidative status was evaluated by quantifying the levels of malondialdehyde (MDA, a lipid peroxidation marker), protein carbonyl (PC), reduced glutathione (GSH), glutathione peroxidase (GPx), and nitric oxide (NO). Spectrophotometric analyses were performed according to established methods: MDA (Nair and Turner 1984), PC (Levine et al. 1990), GSH (Jollow et al. 1974), GPx (Rotruck et al. 1973), and NO (Montgomery and Dymock 1961). All measurements utilized commercial colorimetric kits following the manufacturers’ protocols. Analyses for MDA, GSH, GPx, and NO were performed using kits from Spectrum Biodiagnostics (Cairo, Egypt; catalog numbers MD 25 29, GR 25 11, GP 25 24, and NO 25 33, respectively). The PC assay was conducted using a colorimetric kit from Elabscience (catalog No. E-BC-K117-S). Absorbance readings were obtained using a Shimadzu UV-2401 PC spectrophotometer (Australia).

#### Interleukin − 6 (IL-6) and tumor necrosis factor (TNF-α) assessment in brain tissue

TNF-α and IL-6 concentrations in brain homogenates were quantified using rat-specific ELISA kits (Cat. No. TNF-α: SL0722Ra, IL-6: SL0411Ra, Sunlong Biotechnology Co., LTD, Hangzhou, China) following manufacturer’s protocols.

#### Assessment of acetylcholinesterase (AChE), gamma-aminobutyric acid (GABA), and brain-derived neurotrophic factor (BDNF)

AChE activity, GABA, and BDNF levels in brain homogenates were measured using rat-specific ELISA kits. (Cat. No: SL0027Ra, SL0299Ra, SL1207Ra respectively, Sunlong Biotechnology Co., LTD, Hangzhou, China) according to the manufacturer’s protocols.

#### Evaluation of dopamine and serotonin in brain homogenate

Dopamine (Cat. No. SL0243Ra) and serotonin (Cat. No. SL1046Ra) concentrations were determined via rat-specific ELISA kits (Sunlong Biotechnology Co., LTD, Hangzhou, China) according to manufacturer instructions.

#### Histopathological studies

##### Histopathological analysis

At the end of the experiment, the brain tissues (prefrontal area of the cerebral cortex and the cerebellar cortex) from all rats in the experimental groups were removed and prepared for histological diagnosis. The tissue specimens were immediately fixed in 10% neutral buffered formalin. Then, the fixed tissue samples from the cerebrum and cerebellum were processed, embedded in paraffin blocks and sectioned into 4–5 μm slices using a rotary microtome. After deparaffinization, the sections were stained with hematoxylin and eosin (H&E) and mounted (Suvarna et al. 2013). The prepared slides were examined under a light microscope (Leica DMIL, Leica Microsystems, Germany) at different magnifications to assess tissue morphology and histopathological alterations in a blind manner.

At the end of the experiment, the brain tissues (prefrontal area of the cerebral cortex and the cerebellar cortex) from all rats in the experimental groups were removed and prepared for histological diagnosis. The tissue specimens were immediately fixed in 10% neutral buffered formalin. Then, the fixed tissue samples from the cerebrum and cerebellum were processed, embedded in paraffin blocks and sectioned into 4–5 μm slices using a rotary microtome. After deparaffinization, the sections were stained with hematoxylin and eosin (H&E) and mounted (Suvarna et al. 2013). The prepared slides were examined under a light microscope (Leica DMIL, Leica Microsystems, Germany) at different magnifications to assess tissue morphology and histopathological alterations in a blind manner.

Histopathological scoring of the brain tissues was evaluated as described in previous studies (Bilgiç et al. 2023). Neuronal degeneration and necrosis, perineural space, neurophagia, neuropil vacuolization, inflammatory cell infiltrations, hemorrhagic areas, vascular congestion and perivascular space were estimated for the cerebral tissues. For the cerebellum, changes of shape of Purkinje cells, disappearance of Purkinje cells, degenerated neurons and vascular congestion were scored. The scoring parameters were quantified (three rats/each group) according to their incidence and severity into several grades; no damage: 0, mild damage: 1, mild-moderate damage: 2, moderate damage: 3, moderate-severe damage: 4 and severe damage: 5.

I. Histochemical analysis

The brain tissue sections were stained by toluidine blue (T.B.) and congo red (C.R.) stains to visualize particular histochemical features according to Nallagouni and Reddy (2017). A semi-quantitative scoring system for the different histochemical features were applied, as follows; 0: negative, 1: very weak positive, 2: weak positive, 3: moderate positive, 4: strong positive and 5: very strong positive.

##### Immunohistochemistry analysis

Glial fibrillary acidic protein (GFAP) and inducible nitric oxide synthase (iNOS) immunohistochemistry was performed on paraffin-embedded sections according to Erba et al. (2018). The tissue sections were deparaffinized, rehydrated and incubated in 0.3% hydrogen peroxide solution in methanol at room temperature for 30 min to block endogenous peroxidase activity. Then, the sections were heated in a microwave oven at 720 W for 25 min for antigen retrieval. Incubation with primary antibodies against GFAP (Rabbit mAb, catalog no.: A19058, RRID: AB_2862551) and iNOS (Rabbit pAb, catalog no.: A14031, RRID: AB_2760886) (dil. 1/100 and 1: 50, subsequently) at 4 °C overnight was performed. Followed by, washing with phosphate buffered saline (pH 7.4) and incubation with secondary antibody (dil. 1: 200) and streptavidin/alkaline phosphatase complex (dil. 1: 200) for 30 min at room temperature were done. 3,3′-diaminobenzidine (DAB) was added for 10 min to obtain the staining reaction. Finally, they counterstained with Mayer’s hematoxylin, mounted, and examined by light microscope. Positive brown immunostained cells were counted at 40× magnification for each rat (three rats/each group) in a blinded manner and the mean values were obtained.

#### Statistical analysis

The Shapiro–Wilk test was used to assess the normality of the data distributions. All data sets were confirmed to exhibit a normal distribution, thus supporting the use of parametric tests (One-Way ANOVA). Data are expressed as mean ± SE and analyzed using SPSS v25. One-way ANOVA with Tukey’s post-hoc test assessed intergroup differences (*p* ≤ 0.05). Histopathological data were evaluated using Tukey’s test and Kruskal-Wallis with Dunn’s multiple comparisons.

## Results

### Forced swim test (FST)

**Fig. 1:**
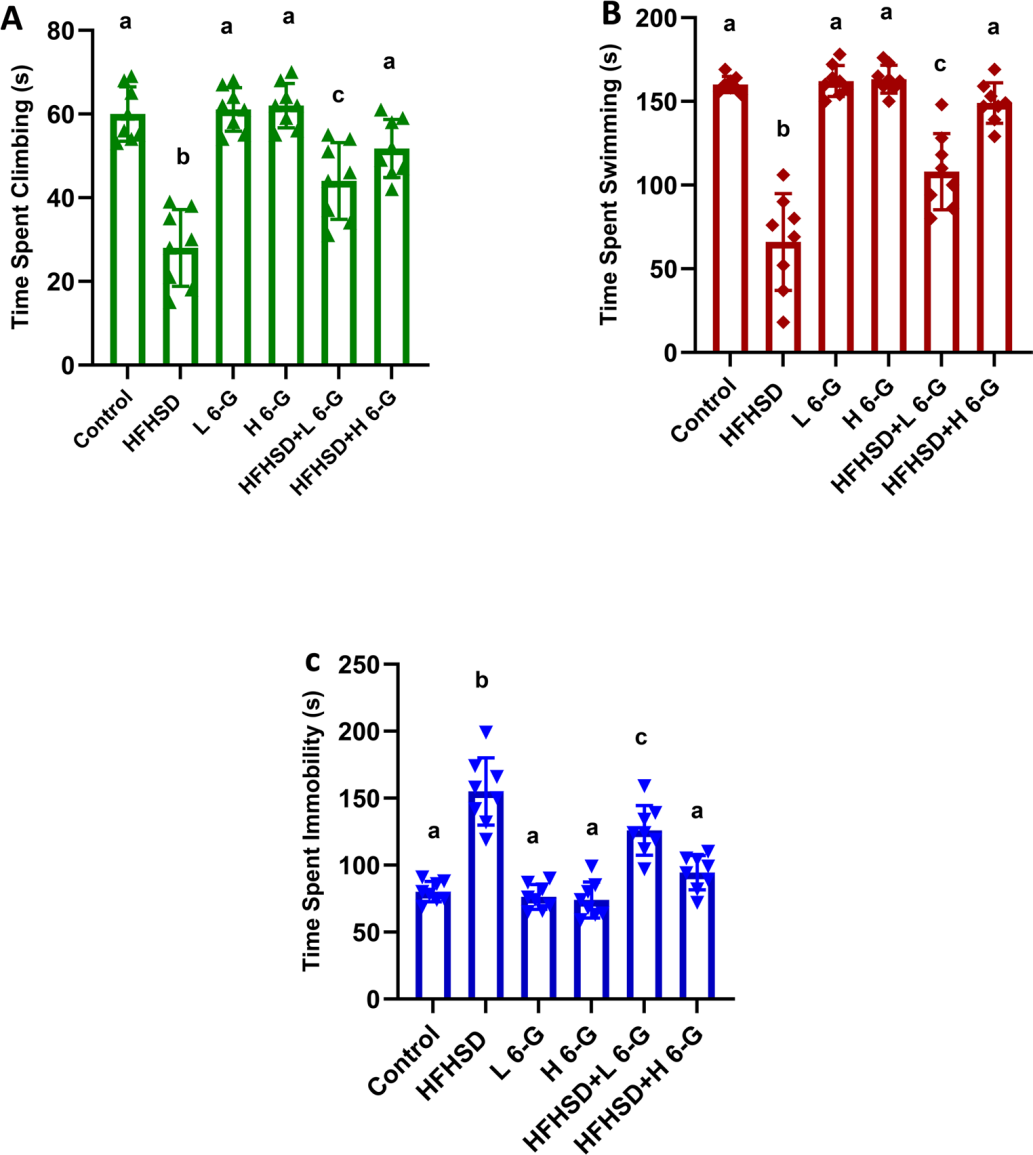
6-Gingerol at doses of 100 and 200 mg/kg ameliorates HFHS diet-induced depressive-like behaviors in the forced swim test. **A**; Time spent in swimming (s). **B**; Time spent in climbing (s). **C**; Time spent in immobility (s). Data are presented as mean ± SEM for n=8 rats per group. All data are presented as Mean ± SEM. Statistical significance was assessed using one-way ANOVA followed by Tukey's post-hoc test. Superscript letters denote statistical differences within each row: values sharing identical letters (e.g., a or b) are not significantly different (p>0.05). Distinct letters indicate a statistically significant difference (p<0.05) between the groups. HFHSD: high fat high sucrose diet group; L 6-G: Low dose 6 gingerol group; H 6-G: high dose 6 gingerol group; HFHSD+ L 6-G: high fat high sucrose diet group+ Low dose 6 gingerol group; HFHSD+ H 6-G: high fat high sucrose diet group+ High dose 6 gingerol group

The HFHS diet group exhibited significant (*p* < 0.05) depressive-like behaviors, showing a 53% reduction in climbing time and 59% reduction in swimming time compared to normal controls, along with a 98% increase in immobility time. Treatment with high-dose 6-gingerol significantly (*p* < 0.05) reversed these effects, increasing climbing time by 79% and swimming time by 109% while decreasing immobility time by 37% compared to the HFHS group. The low-dose group showed intermediate improvements, demonstrating a dose-dependent therapeutic effect. These results indicate that 6-gingerol effectively mitigates HFHS diet-induced behavioral despair, with the high dose restoring activity levels close to normal control values (Fig. 1).

### Tail suspension test (TST)

**Fig. 2:**
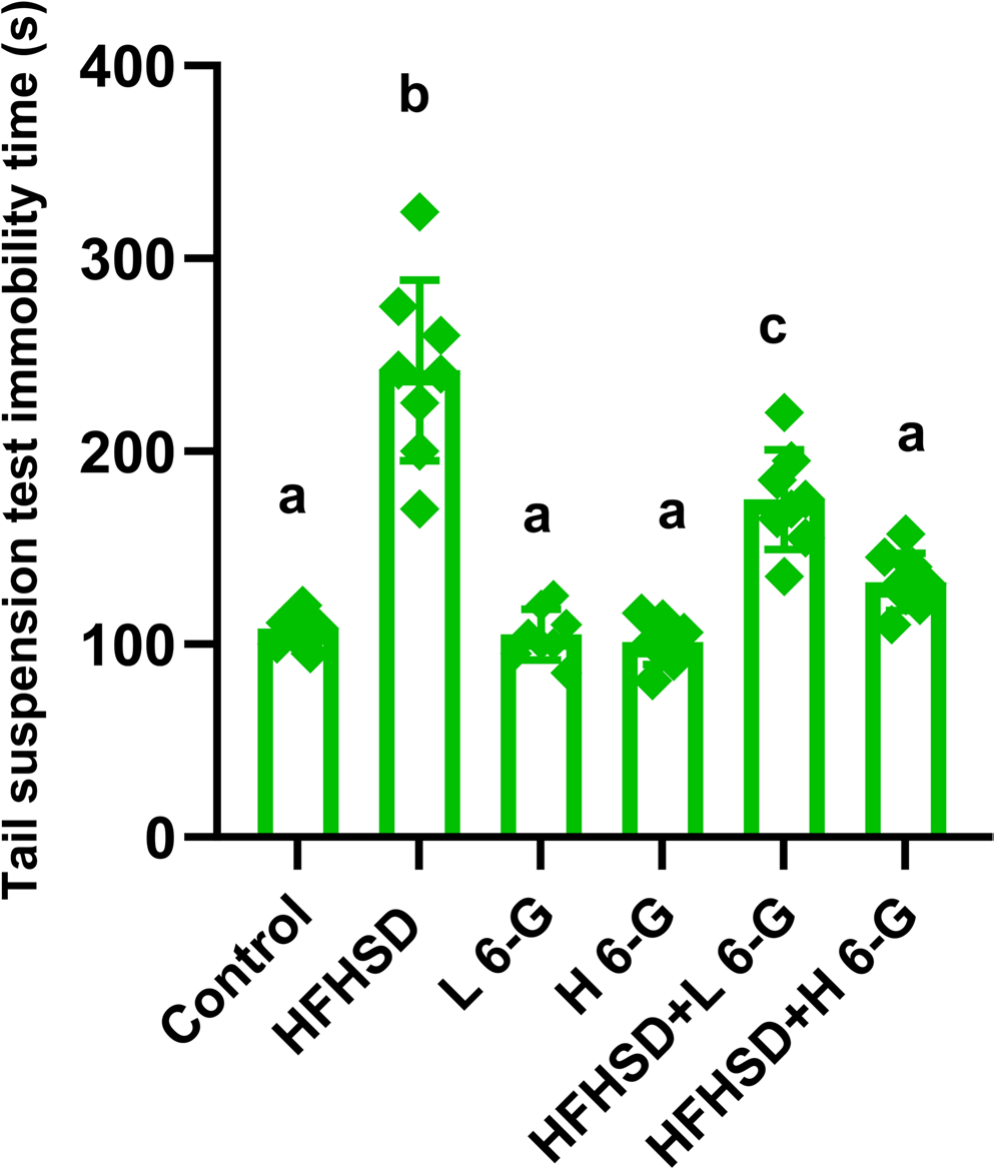
6-Gingerol at doses of 100 and 200 mg/kg ameliorates HFHS diet- Induced Behavioral Despair in the Tail Suspension Test (s). Data are presented as mean ± SEM for *n* = 8 rats per group. All data are presented as Mean ± SEM. Statistical significance was assessed using one-way ANOVA followed by Tukey’s post-hoc test. Superscript letters denote statistical differences within each row: values sharing identical letters (e.g., ^a^ or ^b^) are not significantly different (*p* > 0.05). Distinct letters indicate a statistically significant difference (*p* < 0.05) between the groups. HFHSD: high fat high sucrose diet group; L 6-G: Low dose 6 gingerol group; H 6-G: high dose 6 gingerol group; HFHSD + L 6-G: high fat high sucrose diet group + Low dose 6 gingerol group; HFHSD + H 6-G: high fat high sucrose diet group + High dose 6 gingerol group

As shown in Fig. 2, the HFHS diet group demonstrated significant (*p* < 0.05) depressive-like behavior, exhibiting a 2.2-fold increase in immobility time compared to normal controls. Treatment with 6-gingerol significantly (*p* < 0.05) reduced immobility time in a dose-dependent manner, with the high dose showing a 46% reduction compared to the HFHS group. While the low dose produced a 28% decrease in immobility time, only the high dose restored values to near-normal levels.

### Nutritional parameters

As shown in Table (2), the HFHS diet group exhibited significant (p < 0.05) increases in final body weight (1.58-fold), body weight gain (2.12-fold), feed efficiency ratio (2.15-fold), and relative brain weight (1.39-fold) compared to normal controls. Water intake also increased significantly (p < 0.05) by 1.27-fold in HFHS-fed rats. Treatment with low-dose 6-gingerol partially reversed these effects, reducing body weight gain by 28.8% and feed efficiency by 32.1% versus the HFHS group. High-dose 6-gingerol showed greater efficacy, normalizing final body weight and body weight gain to levels statistically indistinguishable (p< 0.05) from normal controls, while reducing feed efficiency by 46.4% and water intake by 13.2% compared to HFHS rats. Notably, both doses significantly (p < 0.05) restored relative brain weight to control levels.

**Table 2:** Nutritional parameters among in the different experimental groups

parameters	Normal control group	HFHS Diet group	Low dose 6-Gingerol group	High dose 6-Gingerol group	HFHS + Low dose 6-Gingerol group	HFHS + High dose 6-Gingerol group	F-value
Initial body weight (g)	120.3 ± 3.12^a^	120.1 ± 2.99 ^a^	120.2 ± 3.05 ^a^	120.4 ± 3.15 ^a^	120.5 ± 3.11 ^a^	120.4 ± 3.41 ^a^	0.002
Final body weight (g)	250.83 ± 7.12 ^a^	396.38 ± 7.87 ^b^	248.17 ± 6.87 ^a^	245.83 ± 7.05 ^a^	317.27 ± 6.42 ^c^	274.27 ± 6.34 ^a, c^	72.320
Body mass index (g/cm ^2^)	0.462 ± 0.021 ^a^	0.763 ± 0.011 ^b^	0.458 ± 0.019 ^a^	0.455 ± 0.021 ^a^	0.60 ± 0.015	0.511 ± 0.014	49.875
Body weight gain (g)	130.53 ± 4.25 ^a^	276.28 ± 5.34 ^b^	127.97 ± 4.12 ^a^	125.13 ± 4.22 ^a^	196.77 ± 4.84 ^c^	153.87 ± 4.57 ^a^	168.010
Total food intake (g)	998.18 ± 9.13 ^a^	974.81 ± 7.21^a^	995.45 ± 8.45 ^a^	999.12 ± 8.80 ^a^	984.21 ± 7.53 ^a^	999.31 ± 8.22 ^a^	1.499
Feed efficiency ratio	0.13 ± 0.012 ^a^	0.28 ± 0.01 ^b^	0.128 ± 0.011 ^a^	0.125 ± 0.012 ^a^	0.19 ± 0.01 ^c^	0.15 ± 0.01 ^a^	30.873
Total water intake (ml)	2230.5 ± 18.54 ^a^	2826.60 ± 33.56 ^b^	2225.8 ± 19.12 ^a^	2218.3 ± 18.95 ^a^	2607.7 ± 25.42 ^c^	2452.60 ± 21.33 ^a^	114.923
Relative brain weight	0.783 ± 0.02 ^a^	1.088 ± 0.02 ^b^	0.778 ± 0.02 ^a^	0.770 ± 0.02 ^a^	0.740 ± 0.02 ^c^	0.794 ± 0.02 ^a^	42.176

All data are presented as Mean ± SEM. Statistical significance was assessed using one-way ANOVA followed by Tukey’s post-hoc test. Superscript letters denote statistical differences within each row: values sharing identical letters (e.g., ^a^ or ^b^) are not significantly different (*p* > 0.05). Distinct letters indicate a statistically significant difference (*p* < 0.05) between the groups

### Metabolic parameter 

As shown in Table (3) show that The HFHS diet group exhibited significant (p < 0.05) elevations in lipid parameters compared to the normal group, with 3.7-fold higher TC, 3.6-fold higher TG, and 12.4-fold higher LDL-C, alongside reduced HDL-C (29%). Treatment with 6-gingerol significantly (p < 0.05) mitigated these alterations in a dose-dependent manner: the high dose reduced TC (59%), TG (55%), and LDL-C (57%) versus the HFHS group, while restoring HDL-C to near-normal levels (29%). Similarly, HFHS-induced hyperglycemia (1.9-fold) and insulin resistance (3.2-fold) were significantly (p < 0.05) ameliorated by high-dose 6-gingerol (glucose 42%, IR 58%). Inflammatory markers (hs-CRP, leptin) followed a comparable trend, with the high dose normalizing hs-CRP (54%) and leptin (55%) to near-baseline levels. These findings demonstrate 6-gingerol’s significant (p < 0.05) protective effects against HFHS-induced metabolic dysfunction.

**Table 3:** Effects of 6-Gingerol on metabolic parameters in different experimental groups

Parameters	Normal control Group	HFHS Diet Group	Low dose 6-Gingerol group	High dose 6-Gingerol group	HFHS + Low dose 6-Gingerol group	HFHS + High dose 6-Gingerol group	F-value
TC (mg/dL)	79.82 ± 1.23ᵃ	297.32 ± 4.52ᵇ	80.21 ± 1.35ᵃ	77.65 ± 1.40ᵃ	189.59 ± 3.12ᶜ	120.81 ± 2.82ᵈ	1068.520
TG (mg/dL)	67.20 ± 1.45ᵃ	201.90 ± 3.64ᵇ	65.88 ± 1.58ᵃ	65.41 ± 1.62ᵃ	157.94 ± 3.11ᶜ	118.78 ± 2.73ᵈ	527.865
HDL-C (mg/dL)	45.35 ± 1.73ᵃ	32.23 ± 1.04ᵇ	46.12 ± 1.81ᵃ	46.58 ± 1.85ᵃ	36.45 ± 1.51ᶜ	41.57 ± 1.69^a, c^	13.074
LDL-C (mg/dL)	17.93 ± 1.28ᵃ	222.03 ± 4.31ᵇ	16.51 ± 1.41ᵃ	15.87 ± 1.45ᵃ	158.60 ± 3.85ᶜ	94.51 ± 3.53ᵈ	888.874
VLDL-C (mg/dL)	17.44 ± 0.36ᵃ	62.38 ± 0.93ᵇ	17.11 ± 0.40ᵃ	16.98 ± 0.42ᵃ	41.67 ± 0.65ᶜ	28.36 ± 0.44ᵈ	1034.320
Non-HDL-C (mg/dL)	34.47 ± 1.52ᵃ	265.09 ± 4.11ᵇ	32.09 ± 1.67ᵃ	31.07 ± 1.72ᵃ	153.14 ± 3.40ᶜ	80.24 ± 2.99ᵈ	1164.990
TC/HDL-C	1.76 ± 0.22ᵃ	9.22 ± 0.67ᵇ	1.74 ± 0.24ᵃ	1.67 ± 0.25ᵃ	5.20 ± 0.71ᶜ	2.98 ± 0.46^a^	40.455
glucose (mg/dl)	73.71 ± 2.41ᵃ	139.65 ± 3.68ᵇ	72.58 ± 2.55ᵃ	71.83 ± 2.61ᵃ	103.71 ± 2.61ᶜ	81.02 ± 2.11^a^	99.205
Insulin (mU/L)	13.40 ± 1.12ᵃ	22.44 ± 1.57ᵇ	12.92 ± 1.23ᵃ	12.61 ± 1.26ᵃ	19.27 ± 1.41ᶜ	16.13 ± 1.11ᵈ	9.541
IR	2.43 ± 0.81ᵃ	7.74 ± 1.04ᵇ	2.32 ± 0.89ᵃ	2.21 ± 0.91ᵃ	4.93 ± 0.66ᶜ	3.22 ± 0.75^a^	6.538
hs-CRP (ng/ml)	2.93 ± 0.51ᵃ	7.81 ± 0.88ᵇ	2.79 ± 0.56ᵃ	2.71 ± 0.58ᵃ	4.99 ± 0.61ᶜ	3.58 ± 0.58^a^	9.947
Leptin (ng/ml)	24.21 ± 2.58ᵃ	65.37 ± 4.21ᵇ	23.58 ± 2.79ᵃ	23.12 ± 2.85ᵃ	42.96 ± 2.79ᶜ	29.44 ± 2.63^a^	30.646

All data are presented as Mean ± SEM. Statistical significance was assessed using one-way ANOVA followed by Tukey’s post-hoc test. Superscript letters denote statistical differences within each row: values sharing identical letters (e.g., ^a^ or ^b^) are not significantly different (*p* > 0.05). Distinct letters indicate a statistically significant difference (*p* < 0.05) between the groups

### Oxidative stress/inflammatory parameters

The HFHS diet group exhibited significant (p < 0.05) oxidative damage in both cerebrum and cerebellum, with 3.7-fold higher MDA, 2.2-fold higher PC, and 3.1-fold higher NO versus the normal group, alongside 70% depletion of GSH and 67% reduction in GPx activity. Neuroinflammation was markedly elevated, with 3.7-fold higher IL-6 and 3.3-fold higher TNF-α. Treatment with high-dose 6-gingerol significantly (p < 0.05) reversed these effects, reducing MDA (54% in cerebrum, 46% in cerebellum), PC (44% in cerebrum, 41% in cerebellum), and NO (48% in cerebrum, 48% in cerebellum) compared to the HFHS group. Antioxidant defenses were restored, with GSH increasing 2.7-fold and GPx activity 2.7-fold in the high-dose group. Pro-inflammatory cytokines were also significantly (p< 0.05) suppressed (IL-6 67%, TNF-α 62%), nearly normalizing to control levels (Table 2). The low dose showed intermediate effects, while the high dose demonstrated near-complete neuroprotection, highlighting its therapeutic potential against HFHS-induced brain injury (Table 4).

**Table 4:** Neuroprotective effects of 6-Gingerol on oxidative stress, antioxidants, and neuroinflammation in HFHS Diet-induced brain injury among the different studied groups

Brain Tissues	Parametes	Normal control Group	HFHS Diet Group	Low dose 6Gingerolgroup	High dose 6Gingerolgroup	HFHS +Low dose 6-Gingerolgroup	HFHS +High dose 6-Gingerolgroup	F-value
Cerebrum	MDA(nmol/mg tissue)	5.29±1.50ᵃ	19.52±1.11ᵇ	5.01 ± 1.58ᵃ	4.87 ± 1.62ᵃ	14.21±1.09ᶜ	9.04±1.23ᵃ	19.318
	PC(nmol/mg tissue)	24.31±1.46ᵃ	54.48±1.38ᵇ	23.58 ±1.53ᵃ	22.95 ± 1.59ᵃ	39.67±1.51ᶜ	30.25±1.22ᵃ	73.611
	NO(nmol/mg tissue)	5.60±1.47ᵃ	17.24±1.11ᵇ	5.32 ± 1.55ᵃ	5.18 ± 1.60ᵃ	14.54±1.18ᶜ	8.91±0.98ᵃ	15.352
	GSH(nmol/mg tissue)	20.28±0.57ᵃ	6.17±0.29ᵇ	20.82 ± 0.60ᵃ	21.24 ± 0.62ᵃ	11.90±0.39ᶜ	16.72±0.55ᵃ	136.383
	GPx(U/mg tissue)	46.13±0.44ᵃ	15.38±0.64ᵇ	47.25 ± 0.47ᵃ	47.91 ± 0.49ᵃ	32.12±0.47ᶜ	41.07±0.56ᵃ	604.207
	IL-6(pg/mg tissue)	261.13±4.32ᵃ	966±8.62ᵇ	253.45 ±4.55ᵃ	248.12 ±4.65ᵃ	481.31±5.56ᶜ	320.0±4.38ᵃ	2552.760
	TNF-α(pg/mg tissue)	240.22±4.11ᵃ	792.0±6.91ᵇ	233.01 ±4.32ᵃ	228.50 ±4.45ᵃ	379.16±5.11ᶜ	299.11±4.05ᵃ	1964.900
Cerebellum	MDA(nmol/mg tissue)	4.42±1.15ᵃ	15.47±0.99ᵇ	4.20 ± 1.21ᵃ	4.08 ± 1.25ᵃ	11.18±1.23ᶜ	8.31±1.15ᵃ	15.963
	PC(nmol/mg tissue)	19.45±1.31ᵃ	42.79±1.61ᵇ	18.87 ± 1.38ᵃ	18.36 ± 1.43ᵃ	33.60±1.43ᶜ	25.11±1.31ᵃ	49.102
	NO(nmol/mg tissue)	6.45±1.75ᵃ	18.71±1.11ᵇ	6.12 ± 1.83ᵃ	5.96 ± 1.89ᵃ	15.11±0.99ᶜ	9.79±1.08ᵃ	13.043
	GSH(nmol/mg tissue)	22.34±0.61ᵃ	7.40±0.32ᵇ	22.91 ± 0.64ᵃ	23.35 ± 0.66ᵃ	12.85±0.42ᶜ	17.73±0.53ᵃ	141.937
	GPx(U/mg tissue)	46.13±0.44ᵃ	15.38±0.64ᵇ	47.25 ± 0.47ᵃ	47.91 ± 0.49ᵃ	32.12±0.47ᶜ	41.07±0.56ᵃ	604.207
	IL-6(pg/mg tissue)	259.86±4.08ᵃ	909±7.58ᵇ	252.31 ±4.29ᵃ	247.18 ±4.42ᵃ	386.61±4.15ᶜ	316.25±3.89ᵃ	2750.250
	TNF-α(pg/mg tissue)	239.10±3.88ᵃ	789.03±6.8ᵇ	232.12 ±4.08ᵃ	227.78 ±4.22ᵃ	426±4.85ᶜ	300.79±4.11ᵃ	2114.040

All data are presented as Mean ± SEM. Statistical significance was assessed using one-way ANOVA followed by Tukey's post-hoc test. Superscript letters denote statistical differences within each row: values sharing identical letters (e.g., ^a^ or ^b^) are not significantly different (*p*>0.05). Distinct letters indicate a statistically significant difference (*p*<0.05) between the groups

### Neurochemical parameters

The HFHS diet group showed significant (p < 0.05) neurochemical alterations, including 3.8-fold lower BDNF, 3.0-fold lower AChE, 2.7-fold higher GABA, and 1.6–1.8-fold lower serotonin and dopamine versus the normal group in both brain regions. Treatment with high-dose 6-gingerol significantly (p < 0.05) reversed these effects, restoring BDNF to near-normal levels (↑3.1-fold vs. HFHS), normalizing AChE activity (↑2.6-fold), and reducing GABA (↓47%). Monoamine neurotransmitters were similarly rescued, with serotonin and dopamine levels increasing by 54% and 69%, respectively, compared to the HFHS group. The low dose showed intermediate efficacy, while the high dose demonstrated near-complete restoration of neurochemical homeostasis as shown in Table (5), suggesting potent neuroprotective effects against HFHS-induced dysfunction.

**Table 5:** 6-Gingerol modulates neurotrophic factors and neurotransmitters in HFHS Diet-Induced Brain injury

Brain Tissues	Parameters	Normal control Group	HFHS Diet Group	Low dose 6-Gingerol group	High dose 6-Gingerol group	HFHS +Low dose 6-Gingerol group	HFHS +High dose 6-Gingerol group	F-value
Cerebrum	BDNF (µg/g tissue)	90.12±3.87ᵃ	23.68±0.99ᵇ	91.75 ±4.05ᵃ	93.21 ± 4.18ᵃ	49.36±3.21ᶜ	74.04±3.37ᵃ	67.201
	AChE (U/g tissue)	226.21±4.65ᵃ	75.40±3.54ᵇ	230.15 ± 4.88ᵃ	231.87±5.11ᵃ	153.65±3.12ᶜ	198.74±3.81ᵃ	211.699
	GABA (µg/g tissue)	61.31±2.98ᵃ	165.54±3.01ᵇ	59.87 ± 3.13ᵃ	58.52 ± 3.27ᵃ	113.81±3.41ᶜ	87.37±2.89ᵃ	184.799
	Serotonin (ng/g tissue)	417.11±6.30ᵃ	254.26±5.41ᵇ	419.14 ± 6.62ᵃ	420.51±6.88ᵃ	318.63±3.91ᶜ	390.56±4.70ᵃ	143.655
	Dopamine (ng/g tissue)	411.32±5.21ᵃ	228.33±4.73ᵇ	414.88 ± 5.47ᵃ	415.75±5.71ᵃ	297.78±3.22ᶜ	386.11±3.42ᵃ	274.152
Cerebellum	BDNF (µg/g tissue)	87.00±3.65ᵃ	24.85±1.87ᵇ	89.21 ± 3.83ᵃ	90.87 ± 3.98ᵃ	51.22±2.54ᶜ	69.00±2.11ᵃ	71.620
	AChE (U/g tissue)	230.86±4.21ᵃ	82.45±2.24ᵇ	233.52 ± 4.42ᵃ	234.98±4.61ᵃ	160.41±3.21ᶜ	202.71±3.77ᵃ	247.869
	GABA (µg/g tissue)	64.05±3.11ᵃ	163.33±2.99ᵇ	62.48 ± 3.27ᵃ	61.10 ± 3.41ᵃ	115.21±2.76ᶜ	89.15±2.22ᵃ	184.903
	Serotonin (ng/g tissue)	416.0±5.65ᵃ	273.68±4.21ᵇ	417.62 ± 5.93ᵃ	419.24±6.17ᵃ	315.05±3.72ᶜ	391±4.66ᵃ	147.846
	Dopamine (ng/g tissue)	408.34±5.10ᵃ	226.67±4.51ᵇ	411.71 ± 5.36ᵃ	413.37±5.58ᵃ	299.75±3.41ᶜ	381.95±3.20ᵃ	278.184

All data are presented as Mean ± SEM. Statistical analysis was performed using one-way ANOVA followed by Tukey's post-hoc test. Superscript letters denote statistical differences within each row: values sharing identical letters (e.g., ^a^ or ^b^) are not significantly different (*p*>0.05), while distinct letters indicate a statistically significant difference (*p*<0.05) between the compared groups

### Histopathological examination

#### Histopathological findings

**Fig. 3:**
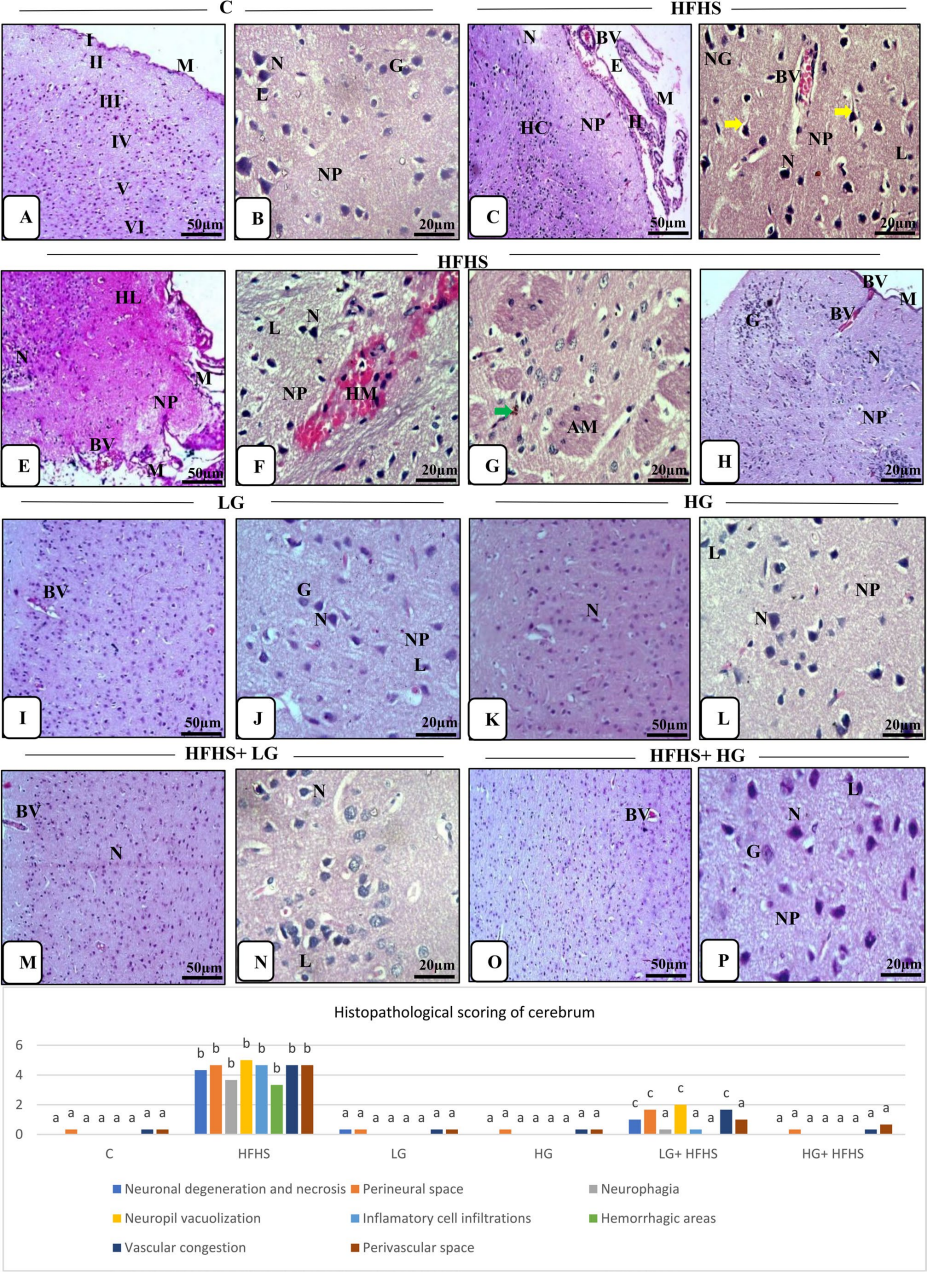
Effect of 6-Gingerol on microscopic examination of cerebral tissues of different treated groups (H&E, Scale bar; x 10: 50 μm & x 40: 20 μm). Data are presented as mean± SEM. Statistical analysis was performed using Tukey's test and Kruskal-Wallis followed by Dunn's multiple comparisons (as applicable). Identical superscript letters indicate a non-significant difference (p>0.05), whereas distinct letters denote a statistically significant difference (p≤0.05). **Control group.** **A:** Normal histological structure: external molecular (I), external granular (II), external pyramidal (III), internal granular (IV), internal pyramidal (V) and multiform layers (VI) and intact meninges (M). **B:** Normal structure. Neurons (N) had large distinct pale staining nuclei and prominent nucleoli with peripheral basophilic Nissl granules, neuroglial cells (L) had small dark nuclei, granule cell (G) with pale open face nucleus and eosinophilic neuropil (NP). **HFHS group.** **C: **Separation of layers of meninges (M) by edema (E) and hemorrhage (H), congested blood vessels (BV), hypercellularity (HC), necrotic area (NA) and vacuolated neuropil (NP). **D:** Prominent necrotic neurons (N) with perineural space, neurofibrillary tangles (yellow arrows), necrotic neuroglial cells (L) with perineural space, neurophagia (NG), vacuolated neuropil (NP) and congested blood vessel (BV). **E:** Separated layers of meninges (M) with congested blood vessels (BV), degenerated neurons (N) with perineural space, hyalinization (HL) and vacuolated neuropil (NP). **F:** Necrotic neurons (N) and neuroglial cells (L) with perineural space, hemorrhagic area (HM) and vacuolated neuropil (NP).** G:** Acidophilic materials (AM) and hemosiderin pigments (green arrow).** H:** Necrotic neurons (N) with perineural space, focal gliosis (GL), inflammatory cells (I), vacuolated neuropil (NP), congested blood vessels (BV) with perivascular spaces of tissue and meninges (M). **LG group. I: **Normal histological structures, congested blood vessel (BV).** J:** Normal neurons (N), neuroglial cell (L) and granule cell (G) within neuropil (NP). **HG group. K: **Normal histology with intact neurons (N). **L:** Normal neurons (N), neuroglial cell (L) and within neuropil (NP). **HFHS +LG group. M:** Improvement in histological structure, some neurons (N) with perineural space, some blood vessels (BV) were congested with perivascular spaces. **N:** Normal neurons, some neurons (N) and neuroglial cells (L) with perineural space.**HG group. O: **Nearly normal histological structure and few congested blood vessels (BV). **P:** Normal neurons (N), neuroglial cell (L) and granule cell (G) and neuropil (NP)
    In contrary, HFHS diet-treated group revealed that prominent multifocal histopathological changes in the form of hypercellularity with discontinuity and separation of layers of meninges when compared with the control group. Signs of degeneration and necrosis were marked on the neurons as deeply shrunken, acidophilic cytoplasm, pyknotic nuclei and loss of their processes and Nissl bodies as well as neurophagia. Also, presence of abnormal neurons with neurofibrillary tangles (flame shaped processes). Granule cells were stained faintly and loss of their nucleoli. Necrotic areas and hyalinization were dispersed in the tissues in some sections. There was a deposition of acidophilic material (amyloid deposition) in some sections. Wide perineural space were seen around neurons and glial cells. Focal astrocytosis, gliosis and inflammatory cell infiltrations specially around blood vessels were seen. Also, there was dilated and congested blood vessels with wide perivascular space as well as vasculitis and vacuolations of their walls were seen among vacuolated neuropil. Variable degrees of edema, hemorrhage with hemosiderin pigments and congested blood vessels were obviously seen in the tissues and meninges (Fig.3 C-H).
    Examination from 6-gingerol-treated groups (low and high) exhibited normal architecture with no apparent pathological abnormalities (Fig.3 I-L).
    In resemblance to the control group, HFHS + LG and HFHS + HG groups revealed an apparent improvement in the histological structures specially in group treated with high dose of 6-gingerol (Fig.3 M-P). Most of the neurons were nearly normal but some of them were necrotic with wide perineural space. Some of the blood vessels showed mild congestion with wide perivascular spaces which more obvious in LG-treated group.
  B **Cerebellar tissues:**
    As evidenced in Fig. 4 A&B, the cerebellar tissues of control group characterized by normal outer cortex of grey matter and inner medulla of white matter. The grey matter was formed from three layers. Firstly, the molecular layer was formed of small scattered superficial stellate cells and large deep basket cells with nerve fibers. Followed by, the Purkinje cell layer that consisted of large pear-shaped Purkinje neurons with prominent nucleolus, Nissl's granules and long apical dendrites as well as they uniformly arranged in one row along the outer margin of the granular layer, in addition to, Bergmann astrocytes and glia cells. The granular layer had vast numbers of very small closely packed granular cells within clear cerebellar islands. The inner medulla contained nerve fibers, supporting neuroglial cells and small blood vessels.

I. ***Histopathological examination***
  A **Cerebral tissues:**
    Microscopically, the cerebral tissues of control group revealed normal histological architecture, consisting of six successive layers from outside to inward with no sharp boundaries; the external molecular, the external granular, the external pyramidal, the internal granular, the internal pyramidal and the multiform. Pyramidal neurons, neuroglia cells and granule cells were visible through the layers. There were many blood vessels with narrow perivascular spaces embedded within acidophilic neuropil that appeared as a mat of neuronal and glial cell processes. The intact meninges were also seen (Fig. 3 A&B).

**Fig. 4:**
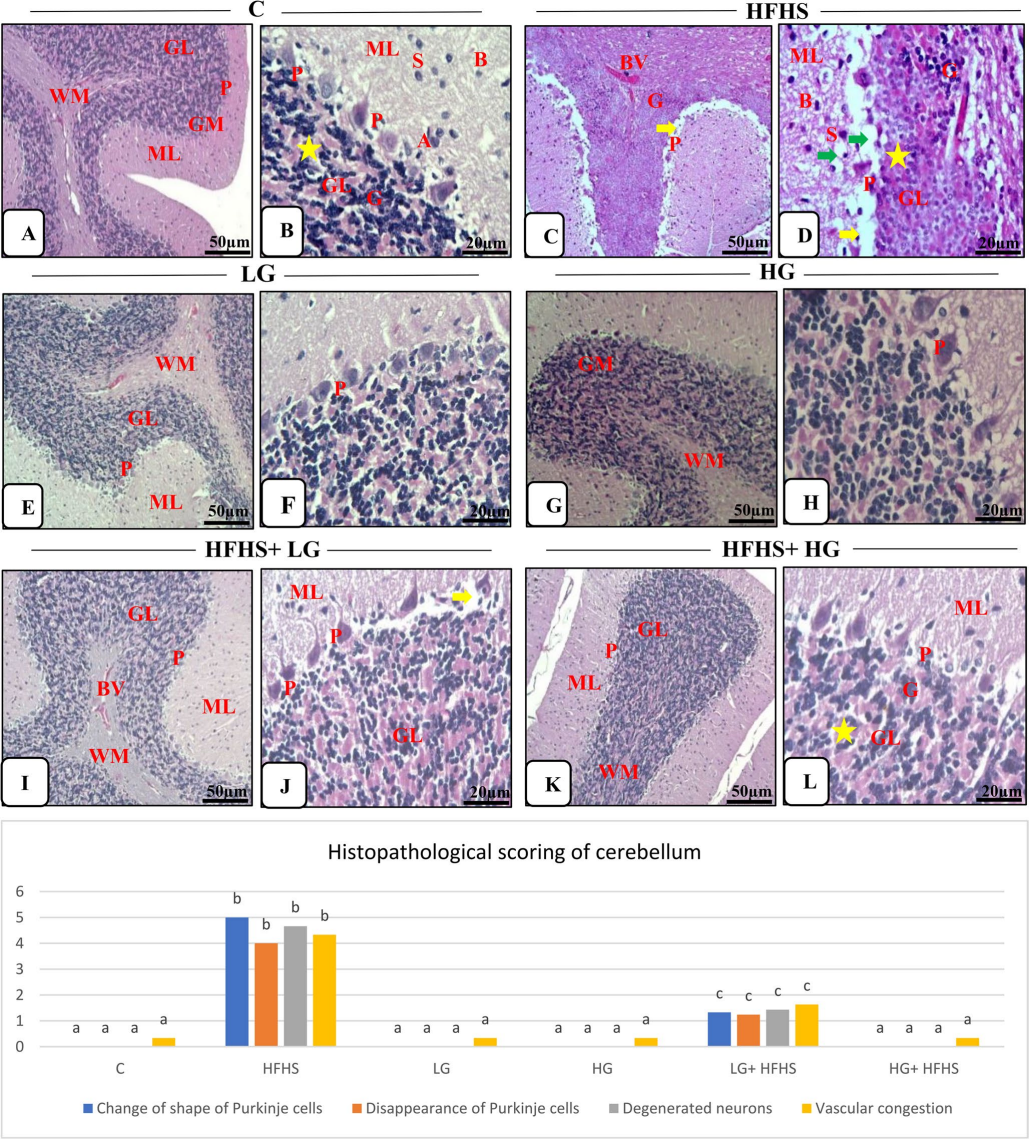
Effect of 6-Gingerol on Microscopic examination of cerebellar tissues of different treated groups (H&E, Scale bar; x 10: 50 μm & x 40: 20 μm). Data are presented as mean± SEM. Statistical analysis was performed using Tukey's test and Kruskal-Wallis followed by Dunn's multiple comparisons (as applicable). Identical superscript letters indicate a non-significant difference (p>0.05), whereas distinct letters denote a statistically significant difference (p≤0.05). **Control group.** **A:** Normal histological structure as molecular layer (ML), Purkinje cell layer (PL), granular cell layer (GL) of grey matter (GM) and white matter (WM). **B:** Stellate cells (S) and basket cells (B) of the molecular layer (ML), Purkinje cells (P) and astrocytes (A) of the Purkinje layer (PL), granular cells (G) and cerebellar islands (asterisk) of the granular layer (GL). **HFHS group.** **C:** Shrunken purkinje cells (P) and most of them were completely lost leaving empty spaces (arrow), necrotic granular cells (G), dilated and congested blood vessels (BV). **D:** Shrunken purkinje cells (P) with dark stained nuclei and empty spaces (yellow arrow), pyknotic basket cells (B) and stellate cells (S) in molecular layer (ML), necrotic granular cells (G) in granular layer (GL), wide cerebellar islands (asterisk) and vacuoles (green arrow). **LG group. I: E: **Normal structure; molecular layer (ML), Purkinje cell layer (PL), granular cell layer (GL) of grey matter and white matter (WM). **F: **Normalstructure with intact purkinje cells (P). **HG group. G: **Normal structure of grey matter (GM) and white matter (WM). **H:** Normalintact purkinje cells (P). **HFHS+LG group.** **I:** Normal arrangement of Purkinje cells of Purkinje layer cell (PL), normal molecular (ML) and granular cell layers (GL) as well as the white matter (WM) with mild congested blood vessels (BV).** J:** Regain of continuous Purkinje cell layer (PL) with normal Purkinje cells (P) but few of them is still shrunken with empty spaces (arrow), normal molecular (ML) and granular cell layers (GL). **HFHS +HG group.** **K:** Normal molecular layer (ML), Purkinje cell layer (PL), granular cell layer (GL) and white matter (WM). **L:** Normal molecular layer (ML), Purkinje cells (P) of the Purkinje layer (PL), granular cells (G) and cerebellar islands (asterisk) of the granular layer (GL)
    While the tissues of HFHS diet-treated group exhibited marked alterations as compared to that of control group. The molecular layer showed pyknotic nuclei of their cells. Marked shrunken purkinje cells with pyknotic nuclei in Purkinje layer, degenerated cells and most of them were completely disappeared leaving empty spaces as well as appearance of numerous astrocytes and glia cells, in addition to, marked disarrangement. The granular cells had clumped appearance with pyknotic nuclei and became thin in some sections with wide cerebellar islands. There was vacuolations in the tissues. Dilated and congested blood vessels were appeared in white matter (Fig. 4C&D).
    Administration of LG or HG- diets, didn’t induce any histopathological changes in the tissues as illustrated in Fig.4 E-H.
    Gingerol had ameliorative effect against neurotoxicity exerted by HFHS diet in LG (Fig.4 I&J) and HG (Fig.4 K&L) groups that caused a somewhat enhancement of cerebellar architecture with normal arrangement of Purkinje cells. The histological picture was more or less similar to control group. Few of Purkinje cells revealed empty spaces and mild congested blood vessels were seen in the low Gingerol-treated group.
  C **Histopathological scoring:**
    The histopathological scoring of the cerebral and cerebellar lesions of H&E examined sections among treated groups was illustrated in Table (6). These observations indicated that the HFHS diet led to extensive structural and cellular brain damage as a result of neuroinflammation and disrupted neurovascular integrity. The histopathological scoring of cerebral tissues, revealed significant neurodegenerative and inflammatory alterations in the HFHS diet-fed group. There was a prominent increase in scores for neuronal degeneration and necrosis, perineural space enlargement, neurophagia, and neuropil vacuolization compared to the control group. Furthermore, elevated levels of scores of inflammatory cell infiltration, vascular congestion, hemorrhagic areas, and perivascular space were evident. The scoring of cerebellar tissues revealed significant structural alterations in the HFHS diet-fed group, including changes in Purkinje cell shape, loss of Purkinje cells, neuronal degeneration, and vascular congestion.

**Table 6:** Different histopathological parameters among treated groups

	C	HFHS	LG	HG	LG+ HFHS	HG+ HFHS	F value
I-Histopathological Scoring A- A-Cerebrum:							
1-Neuronal degeneration and necrosis	0.00±0^a^	4.333±0.333^b^	0.333±0.333^a^	0.00±0^a^	1.00±0.578^c^	0.00±0^a^	94.14
2-Perineural space	0.333±0.333^a^	4.666±0.333^b^	0.333±0.333^a^	0.333±0.333^a^	1.666±0.333^c^	0.333±0.333^a^	82.4
3-Neurophagia	0.00±0^a^	3.666±0.333^b^	0.00±0^a^	0.00±0^a^	0.333±0.333^a^	0.00±0^a^	58.9
4-Neuropil vacuolization	0.00±0^a^	5.0±0^b^	0.00±0^a^	0.00±0^a^	2.0±0.578^c^	0.00±0^a^	112.7
5-Inflamatory cell infiltrations	0.00±0^a^	4.666±0.333^b^	0.00±0^a^	0.00±0^a^	0.333±0.333^a^	0.00±0^a^	94.96
6-Hemorrhagic areas	0.00±0^a^	3.333±0.333^b^	0.00±0^a^	0.00±0^a^	0.00±0^a^	0.00±0^a^	50.4
7-Vascular congestion	0.333±0.333^a^	4.666±0.333^b^	0.333±0.333^a^	0.333±0.333^a^	1.666±0.333^c^	0.333±0.333^a^	82.14
8- Perivascular space	0.333±0.333^a^	4.666±0.333^b^	0.333±0.333^a^	0.333±0.333^a^	1.0±0.578^a^	0.666±0.333^a^	78.9
B- Cerebellum: 1-Change of shape of Purkinje cells	0.00±0^a^	5.00±0^b^	0.00±0^a^	0.00±0^a^	1.33±0.333^c^	0.00±0^a^	108.60
2-Disappearance of Purkinje cells	0.00±0^a^	4.00±0.578^b^	0.00±0^a^	0.00±0^a^	1.24±0.578^c^	0.00±0^a^	70.06
3-Degenerated neurons	0.00±0^a^	4.66±0.333^b^	0.00±0^a^	0.00±0^a^	1.43±0.333^c^	0.00±0^a^	95.02
4-Vascular congestion	0.333±0.333^a^	4.33±0.664^b^	0.333±0.333^a^	0.333±0.333^a^	1.63±0.333^c^	0.333±0.333^a^	70.33
II-Histochemical examination 1- T.B. stain							
A- A- Cerebrum	0.00±0^a^	4.66±0.333^b^	0.00±0^a^	0.00±0^a^	1.00±0.578^c^	0.333±0.333^a^	91.03
B- Cerebellum	0.00±0^a^	4.33±0.667^b^	0.00±0^a^	0.00±0^a^	1.43±0.333^c^	0.00±0^a^	82.62
2- C.R. stain							
A- Cerebrum	0.00±0^a^	4.66±0.333^b^	0.00±0^a^	0.00±0^a^	0.666±0.667^a^	0.333±0.333^a^	93.13
B- Cerebellum	0.00±0^a^	4.0±1^b^	0.00±0^a^	0.00±0^a^	1.0±0.578^a^	0.666±0.333^a^	65.37
III-Immunohistochemistry analysis 1- GFAP							
A- Cerebrum	2.66±0.878^a^	24±2.08^b^	3.66±0.878^a^	3.66±0.878^a^	10.66±0.878^c^	3.66±0.878^a^	1881
B- Cerebellum	2.66±1.2^a^	22±5.29^b^	2.66±0.878^a^	3.66±0.878^a^	4.75±1.2^a,c^	2.66±0.878^a^	1597
2- iNOS							
A- Cerebrum	0.333±0.333^a^	15±2.89^b^	0.333±0.333^a^	0.333±0.333^a^	1.66±0.878^c^	0.666±0.890^a^	933.5
B- Cerebellum	0.00±0^a^	14.33±3.84^b^	0.333±0.333^a^	0.00±0^a^	1.66±0.333^c^	0.333±0.333^a^	836.7
II. Histochemical examination
  Examination of toluidine blue of cerebral and cerebellar tissue sections of different groups showed in Fig. 5 (A-L) and Table (6). The control one, LG and HG groups revealed intact normal stained cells; normal neurons with pale cytoplasm and basophilic Nissl’s granules as well as glial cell and granule cells within the intact neuropil in the cerebrum. The structural layers of the cerebellum had normal intact cells; the Purkinje cells with a central vesicular nucleus and visible Nissl's granules. In contrary, HFHS diet-treated group showed necrotic neurons and Purkinje cells with less Nissl's granules in both cerebrum and cerebellum that detecting necro-degenerative changes of neurons. However, treatment with HFHS diet and gingerol, the tissues revealed normal cells and some of them with less Nissl’s granules specially in low gingerol-treated group.

**Fig. 5:**
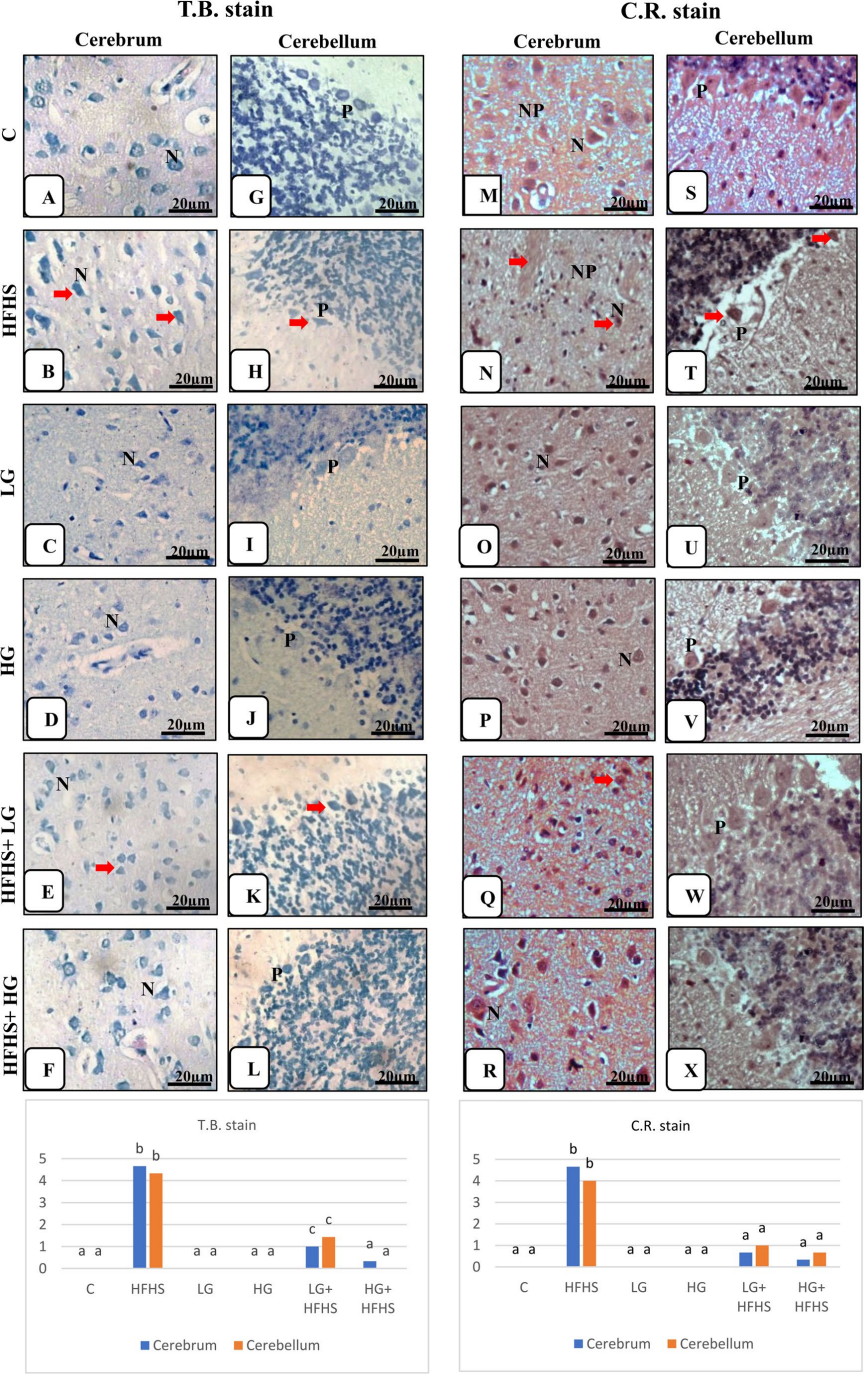
Effect of 6-Gingerol on Microscopic examination of cerebral and cerebellar tissues of different treated groups identified by deep blue stained Nissl's granules (arrows) by Toluidine blue stain (A-L) and deep red positive reaction (arrows) by Congo red stain (M-X) (Scale bar; x 40: 20 μm). Data are presented as mean ± SEM. Statistical analysis was performed using Tukey's test and Kruskal-Wallis followed by Dunn's multiple comparisons (as applicable). Identical superscript letters indicate a non-significant difference (p>0.05), whereas distinct letters denote a statistically significant difference (p≤0.05). **Toluidine blue stain -** **Cerebrum: A:** Intact normal stained neurons with marked basophilic Nissl’s granules (N).** B:** Necrotic neurons with less Nissl's granules (N) and perineural space.**C:** Normal staining intensity. **D:** Nearly normal structure. **E:** Normal staining neurons (N) and some of them were degenerated with less Nissl's granules. **F:** Normal staining neurons with visible Nissl's granules (N). **Toluidine blue stain - Cerebellum: G:** normal intact Purkinje cells with a central vesicular nucleus and visible Nissl's granules (P). **H:** Necrotic Purkinje cells with less Nissl's granules. **I:** Nearly normal Purkinje cells. **J:** Normal staining intensity. **K:** Normal Purkinje cells with Nissl's granules and some of them were degenerated. **L:** Normal Purkinje cells with visible Nissl's granules (P). **Congo red stain -** **Cerebrum: M: **Negative reaction for stain and intact neurons (N) within normal neuropil (NP). **N:** Deeply stained amyloid deposits in degenerated neurons (N) and neuropil (NP). **O:** Normal stained neurons (N). **P: **Normal neurons (N). **Q:** Mild deposition of amyloids in some neurons and normal neuropil. **R:** normal staining neurons (N) and neuropil. **Congo red stain - Cerebellum: S:** Normal stained Purkinje cells (P).**T:** Deeply stained degenerated Purkinje cells (P). **U:** Normal stained Purkinje cells (P).**V:** Normal Purkinje cells (P).**W:** Mild positive reaction of stain of some Purkinje cells (P). **X:** Normal stained Purkinje cells (P)
  In the Congo red-stained tissue sections, amyloid deposits in the affected cells and the neuropil as a dark pink homogenous extracellular distribution as well as irregular shape of neurons were detected in cerebral and cerebellar tissues in HFHS diet-treated group compared with control group. While tissues of low and high gingerol only–treated groups resembled normal control group. Treatment of HFHS diet-treated group with 6-gingerol had appearance of mild deposits of amyloid material specially in LG +HFHS treated group with normal morphology of neurons (Fig. 5 M-X and Table6).
III. Immunohistochemistry analysis:
  Brain tissue sections were stained with GFAP immunostaining, as shown in Fig. 6 (A-L) and Table 6. In the control rats, weak positive GFAP immunoreactivity of the astrocytes in the cerebrum and the cerebellum were seen and they appeared as few dispersed brown cells with network of fine processes extending throughout the tissues. They could be seen surrounding neurons and blood vessels. In contrast, there was strong positive immunostained cells with several thick branched processes and increased in size and number in both the cerebrum and cerebellum in response to the HFHS diet-treated group compering with control group. As a result of astrogliosis, the cells became hypertrophied and increase in number. The gingerol+ HFHS diet treated rats showed mild to moderate positive GFAP immunoreactivity in tissues which was more common in LG+ HFHS treated group. Table (6) showed significantly increasing in GFAP immunoreactivity in HFHS diet-fed group (24.0±2.08, 22.0±5.29) compared to control across the cerebral and cerebellar tissues. While LG + HFHS group exhibited a moderate increase (10.66±0.88). This upregulation indicates enhanced astrocyte activation, consistent with neuro-inflammatory responses associated with metabolic stress induced by the HFHS diet.

**Fig. 6:**
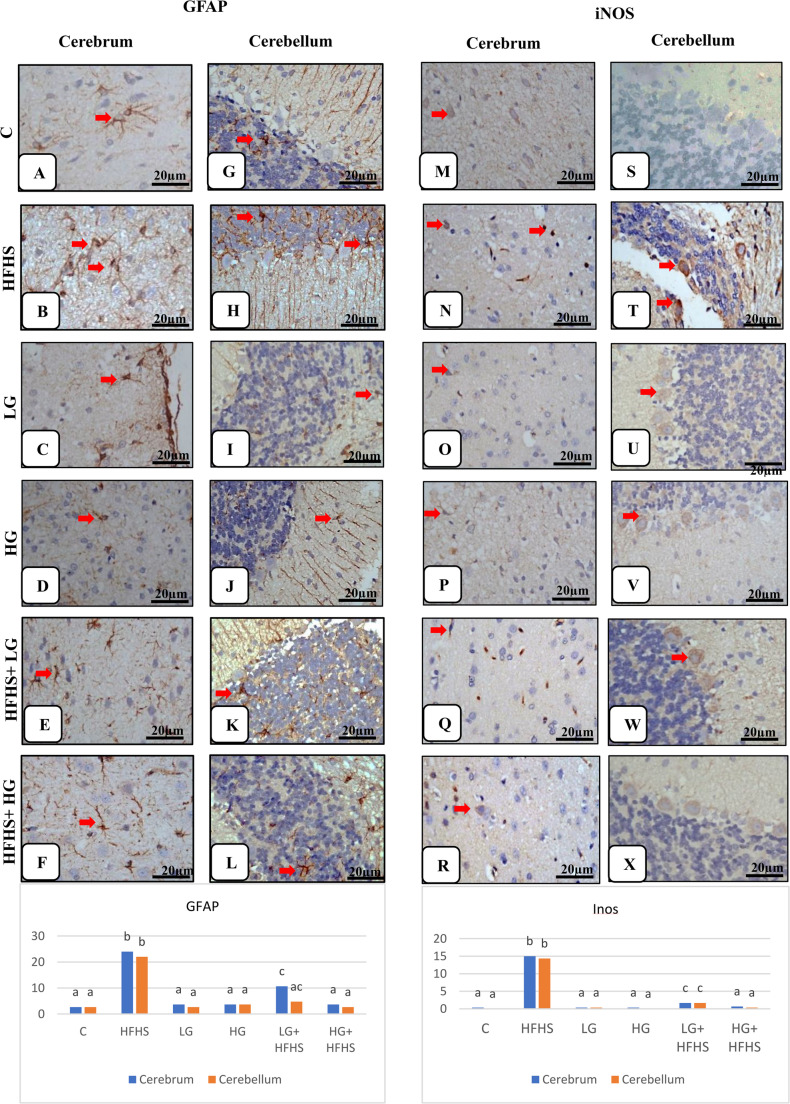
Effect of 6-Gingerol on Microscopic examination of cerebral and cerebellar tissues of different treated groups identified by brown color of immunostained cells by GFAP (A-L) and iNOS (M-X) (arrows) (GFAP and iNOS immunohistochemical staining, Scale bar; x 40: 20 μm). Data are presented as mean ± SEM. Statistical analysis was performed using Tukey's test and Kruskal-Wallis followed by Dunn's multiple comparisons. Identical letters indicate a non-significant difference, whereas distinct letters denote a significant difference at p ≤ 0.05. **GFAP - Cerebrum: A:** Normal weak positive immunoreactivity for GFAP of few astrocytes.** B: **Strong positive immunostained cells with several long thick branched processes. **C: **Weakpositive reaction. **D:** Mild positive immunoreactivity. **E:** Reduced immuno-staining intensity of cells for GFAP. **F:** Mild reaction of some cells. **GFAP - Cerebellum: G:** Weak positive GFAP immunoreactive cells scattered in the Purkinje cell layer and the granular layer. **H:** Intense positive immune expression of astrocytes for GFAP in all layers. **I:** WeakGFAP staining intensity. **J:** Weak positive immunoreaction. **K:** Moderate immune-staining cells. **L:** Weak positive cells for GFAP. **iNOS - Cerebrum: M:** Minimal expression of **i**NOS reaction tissues. **N: **Strong positive immune-reactive cells. **O:**Faintpositive reaction. **P:** Weak iNOS expression.** Q: **Moderate reaction of cells. **R:** Weak immune-staining cells. **iNOS - Cerebellum: S:** Negative immunoreactivity of the cells. **T: **Intensive positive immune expression for iNOS. **U:** Weak positive reaction. **V:** Weak positive immunoreactive cells. **W:** Moderate immune-staining. **X: **Weak immune-reaction.
  The findings illustrated in Fig. 6 (M-X). and Table (6) that showed of the iNOS immune-reactions in the cerebrum and cerebellum tissues among the different experimental groups. iNOS immunoreactivity was no to very weak in the cytoplasm of glia cells and astrocytes in the cerebrum and cerebellum of the control rats. In contrast, the HFHS diet-treated group was exhibited a significant increase in iNOS expression in glia cells and astrocytes under activation, in addition to, neurons of cerebrum and Purkinje and basket cells of cerebellum as well as endothelial cells of the blood vessels as a neuro-inflammation sign. While the 6-gingerol treatment with HFHS diet resulted in a dose-dependent decrease in iNOS expression in neurons.
  As shown in Table 6, the levels of iNOS immunoreactivity in the HFHS diet-fed group were significantly elevated in both the cerebral (15.0 ± 2.89) and cerebellar (14.33 ± 3.84) tissues compared to the control group. This increase reflects heightened inflammatory activity, suggesting that the HFHS diet induces neuro-inflammation across multiple brain regions.

## Discussion

The rising prevalence of obesity and overweight is partly attributed to hypercaloric, palatable diets, which promote excessive weight gain by storing unused calories as adipose tissue. Studies indicate that high-fat, high-sugar diets exacerbate body weight gain (Dourmashkin et al. 2005; Martire et al. 2015). Consistent with prior research (Ginsberg 2006), our study observed altered serum lipid profiles (T-Chol, TG, HDL-C, LDL-C) in HFHS-fed rats. Hypercaloric diets induce postprandial hypertriglyceridemia, hyperglycemia, and elevated free fatty acids, contributing to oxidative stress in obesity models (Feillet-Coudray et al. 2019; Essa et al. 2025).

Leptin, a regulator of metabolism, is elevated in MetS (Ghadge and Khaire 2019). In our study, HFHS-fed rats exhibited increased leptin levels compared to controls, which were dose-dependently reduced by 6-gingerol. Similar findings confirm that 6-gingerol and ginger lower serum leptin in high-fat diet models (Saravanan et al. 2014).

6-Gingerol also improved insulin sensitivity, evidenced by reduced HOMA-IR and fasting blood glucose, likely via adipocytokine regulation. Proper insulin function is crucial for glucose and cholesterol homeostasis (Pihlajamäki et al. 2004). Our findings showed that HFHS-fed rats displayed dyslipidemia (elevated TC, TG; reduced HDL-C), which 6-gingerol attenuated dose-dependently. Additionally, 6-gingerol reduced body weight, aligning with studies linking adipocytokines (leptin, vaspin) to glucose metabolism (Taheri et al. 2020). Our results suggest that 6-gingerol’s hypoglycemic and hypolipidemic effects are partly mediated by leptin modulation.

Reduced caloric intake and body weight in 6-gingerol-treated rats may stem from suppressed appetite, consistent with findings in STZ-induced diabetic rats (Samad et al. 2017). This effect could involve enhanced GLP-1-mediated insulin secretion and skeletal muscle glucose uptake.

In this study, the administration of the HFHS diet increased oxidative stress and pro-inflammatory cytokines (MDA, PC, NO, IL-6, TNF-α) while reducing GPx and GSH in brain tissues (cerebrum, cerebellum). Crucially, the high metabolic burden of the HFHS diet resulted in pronounced oxidative damage in both the cerebrum (central to affective behavior) and the cerebellum (a highly metabolically active region). 6-Gingerol, especially at high dose, significantly reversed these effects. Similar anti-inflammatory effects were observed in diabetic and non-alcoholic steatohepatitis rat models (Almatroodi et al. 2021; Essa et al. 2026). Reduced adiponectin and elevated IL-6 in diabetic patients further support 6-gingerol’s role in mitigating inflammation via metabolic and leptin regulation.

Depression is strongly linked to metabolic disorders like obesity and diabetes (Euesden et al. 2017). MetS, characterized by obesity, insulin resistance, and dyslipidemia (Martins et al. 2019), induced neurotransmitter disturbances and depression-like behaviors in our study, which 6-gingerol ameliorated dose-dependently. Obesity and depression share a bidirectional relationship. Metabolically unhealthy obesity (e.g., with dyslipidemia or insulin resistance) elevates depression risk (Milaneschi et al. 2019), likely via neuroimmune activation (Gregor and Hotamisligil 2011). Elevated IL-6 and CRP in depression (McIntyre et al. 2007) and hypothalamic-pituitary-adrenal axis dysregulation (Southwick et al. 2005) further implicate inflammation in depressive pathophysiology. Chronic glucocorticoid excess impairs glucose uptake, promoting fat accumulation and insulin resistance, which may damage neurons and alter mood (McIntyre et al. 2007).

Severe depression is linked to neurodegenerative processes driven by chronic inflammation, wherein proinflammatory cytokines disrupt critical neurochemical pathways. These cytokines impair monoamine synthesis—specifically serotonin and dopamine—by downregulating tetrahydrobiopterin (BH4), a vital cofactor for tryptophan hydroxylase and tyrosine hydroxylase (Vancassel et al. 2018). Reactive oxygen and nitrogen species further exacerbate this disruption, suppressing norepinephrine synthesis and altering neurotransmitter balance.

Concurrently, chronic inflammation compromises neuroplasticity by interfering with neurotrophic factors such as brain-derived neurotrophic factor Brain-Derived Neurotrophic Factor (BDNF). The resulting damage to nerve cell membranes and reduced repair of dendrites and axons contribute to synaptic dysfunction and neurodegeneration. Moreover, inflammatory cytokines dysregulate dopamine signaling in reward circuits, manifesting as core depressive symptoms—anhedonia, fatigue, and psychomotor retardation (Felger and Lotrich 2013a, b).

Our comprehensive neurochemical analysis provides a mechanistic basis for the depressive-like behaviors observed in HFHS-fed rats by showing severe regional dysregulation. The observed reduction in cerebral serotonin and dopamine levels directly correlates with the core symptoms of depression, including anhedonia, psychomotor retardation, and despair, as evidenced by increased immobility in the FST and TST. The serotonergic system, crucial for mood regulation, is particularly vulnerable to diet-induced inflammation and oxidative stress, which can suppress the synthesis of key monoamines (Felger and Lotrich 2013a, b). Furthermore, the significant elevation in AChE activity in the cerebrum suggests heightened cholinergic signaling, which has been implicated in negative feedback on dopamine release and is associated with behavioral despair (Mineur et al. 2013; Miret et al. 2013).

Notably, parallel dysregulation was found in the cerebellum, a region increasingly recognized for its role in affective processing. The disruption of GABAergic tone and neurotrophic support (BDNF) in this region likely contributes to the emotional and cognitive inflexibility characteristic of depressive states (Laricchiuta et al. 2018).

Ginger and its active constituents can influence central nervous system 5-HT metabolism and function through various mechanisms, such as enhancing its synthesis, reducing its degradation or release, or blocking its receptors (Bano et al. 2021) demonstrated that ginger extract exhibits anti-anxiety effects in anxious behavior models, potentially due to increased serotonin synthesis and altered tryptophan metabolism and distribution. A decrease in anxiety may stem from ginger’s impact on neurotransmitters like 5-hydroxytryptamine (5-HT) and GABA. According to Perveen et al. (2009), ginger elevates 5-HT levels, thereby reducing anxiety.

It has been indicated that anxiety and depressive disorders, rather than being distinct, share common symptoms and pathogenic mechanisms. In the current study, the forced swimming test and tail suspension test revealed depressive behavior in rats fed a high-fat, high-sucrose diet. Co-administration of 6-gingerol increased swimming time and reduced immobility time in both FST and TST, suggesting antidepressant-like activity of this antioxidant. Monoamine neurotransmitters like serotonin and dopamine are crucial in mediating depressive behaviors. It is well-established that swim/immobility behaviors influence serotonergic signaling in the brain, leading to increased synaptic transmission that ultimately alters responses from immobility to swimming and climbing in the FST.

The efficacy of 6-gingerol stems from its ability to resolve the upstream drivers of this widespread neurochemical dysfunction. the restoration of cerebral and cerebellular BDNF by 6-gingerol is of critical importance, as this neurotrophin supports synaptic plasticity, neuronal survival, and the functional integrity of circuits governing mood and reward. The concomitant reduction in GFAP and iNOS indicates that 6-gingerol’s primary action may be the suppression of neuroinflammation and oxidative stress, which are upstream drivers of this widespread neurochemical dysfunction (Duman and Monteggia 2006). By quelling this central inflammatory milieu, 6-gingerol creates a permissive environment for the restoration of monoaminergic, cholinergic, and neurotrophic systems, thereby reversing the behavioral phenotype of depression (Zhang et al. 2018).

6-gingerol, isolated from ginger rhizome oil, has been shown to affect neurotransmission in snails and possess neuroprotective effects in rodents (Shen et al. 2024) demonstrated that gingerol-enriched ginger (GEG) supplementation improved anxio-depressive behaviors in rats with diabetic neuropathy. This indicates that 6-gingerol and related compounds can influence mood, even in the presence of metabolic complications. The study also reported that GEG reduced neuroinflammation in these rats.

In the present work, there was hypercellularity and necro-degenerative alterations of neurons with meninges discontinuity and separation in the cerebral and cerebellar tissues in HFHS diet-treated group when compared with the control group. This was in accordance with the previous study of (Samad et al. 2023). The neuronal changes were leading to the formation of reactive oxygen species (ROS) and nitrogen species with defects of antioxidant defense system which caused DNA damage and neuronal cell death (Oruc and Uner 2002).

Our results aligned with the observation of (Zetterberg et al. 2010), who observed the presence of abnormal neurons with neurofibrillary tangles, deposition of amyloid proteins (amyloid-beta, Aβ) with inflammatory cell infiltrations in rats fed on HFHS which were characteristic for Alzheimer’s disease (AD). The later is a neurodegenerative disease with progressive degradation of learning, memory impairment, and cognition decline symptom (Niu et al. 2016). Additionally, (Takeda et al. 2013) discussed the role of inflammatory factors as a results of diet as they released to the blood plasma and enter the brain leading to brain inflammation and non-cognitive symptoms, such as depression, agitation, and psychosis.

Astrocytosis, gliosis and inflammatory cell infiltrations in the current work were parallel to the results of (Zedan 2023) who reported that the astrocytes caused inactivating ROS. While glial cells had a metabolic support to neurons, and offering protection against oxidative stress (Baydas et al. 2003). Inflammatory factors triggered by hyperglycemia activated glial cells, which in turn release additional pro-inflammatory mediators and progression of neurodegenerative diseases. Moreover, activated microglial cells produced amyloid precursor protein (APP), which gave rise to neurotoxic amyloid proteins implicated in these conditions (Takeda et al. 2013).

Intercellular vacuolations were observed in the examined tissues, consistent with the findings of (Zedan 2023), who attributed them to the lipid peroxidation and damage to both the cell and organelle membranes.

The histopathological scoring of observable lesions, with or without treatment showing notable improvement in tissue integrity and reduced lesion severity, was consistent with the findings reported by (Esrefoglu et al., 2014).

Toluidine blue stain is commonly used to evaluate neuronal integrity and visualize Nissl granules in the brain tissue. In the present study, HFHS diet-treated groups showed marked neuronal damage with less Nissl granules distribution that indicating neurodegeneration which consistent with (Haq and AlAmro 2019). Congo red-stained tissue sections revealed prominent amyloid deposits in the tissues of the HFHS diet-treated group. Similar findings were reported by (Abdel-Salam et al. 2015), who observed comparable amyloid accumulation as a result of neurodegeneration induced by AlCl₃ toxicity.

In the current study, the HFHS diet-treated group exhibited a significant increase in GFAP-positive astrocytes, likely resulting from enhanced astrocyte proliferation as a results of oxidative stress, in line with the findings of Mota et al. (2023a, b). On the other hand, iNOS was more highly expressed in the brain tissues of HFHS diet-treated group compared with control group. This finding was align with the elevated iNOS levels reported in sodium nitrite-treated rats by (Özen et al. 2014).

This study demonstrated that examination of tissue sections from the HFHS diet-treated group receiving 6-gingerol showed a noticeable reversal of most HFHS diet-induced histopathological changes, particularly in the high dose-treated group. Our findings were mimic with another study by (Hussein et al. 2017). Ginger had been documented to alleviate hyperglycemia and hyperlipidemia and was known for anti-inflammatory, antioxidant, antimicrobial, and anticancer effects as well as its ability to scavenge free radicals. In addition to, ginger exhibits anti-amyloidogenic properties and can inhibit astrocyte over activation, both of which are key factors in the development of neurodegenerative diseases so ginger may help alleviate cognitive impairment symptoms (Arcusa et al. 2022). Also, there was a notable improvement in neuronal morphology and restoration of Nissl granules, as evidenced by enhanced toluidine blue staining which was resemble to results of Haq and AlAmro (2019). CR staining showed a noticeable decrease in amyloid deposition in these groups which was in line with (Bassiony et al. 2015).

This study demonstrated the reduction of GFAP expression that confirmed by the previous reports of (Essawy et al. 2023), who showed that melatonin exerted protective effects against tartrazine-induced neurotoxicity in rats. Furthermore, 6-gingerol reduced the expression of iNOS, indicating its potential anti-inflammatory effect. Similar findings were reported by (El-Akabawy and El-Kholy 2014), who observed a significant decrease in iNOS levels in diabetic rats treated with ginger. limitations of the work: despite demonstrating a robust neuroprotective effect, this study has limitations. It was conducted exclusively on male rats, meaning the findings may not be generalizable to females due to known sex differences in metabolic and affective regulation. Furthermore, the research is constrained by its rodent model design, limiting the direct translational applicability of the dose-response and long-term efficacy to human clinical settings.

## Conclusion

This study demonstrates that 6-gingerol, particularly at the higher dose (200 mg/kg), significantly counteracted HFHS diet-induced metabolic and neurobehavioral dysfunction. Both doses (100 and 200 mg/kg) improved lipid profiles, insulin sensitivity, and oxidative/inflammatory markers, but the higher dose showed superior efficacy in attenuating depressive-like behaviors (reduced immobility in FST/TST) and restoring neurotransmitter balance (↑ serotonin, dopamine, GABA; ↓ AChE). Moreover, BDNF showed improvements associated with dose-dependent reductions in neuroinflammation (GFAP, iNOS) and neurodegeneration (amyloid deposits, neuronal vacuolation). These findings underscore 6-gingerol’s potential as a neuroprotective agent against diet-induced metabolic syndrome and depression, with effects mediated through antioxidant, anti-inflammatory, and neurotransmitters modulation.

## Data Availability

The authors confirm that the data supporting the findings of this study are available within the article.

## Abbreviations

AChE: Acetylcholinesterase
AIN-93: American Institute of Nutrition 1993 diet formulation
ARC-ARRI: Agriculture Research Centre - Animal Health Research Institute
BDNF: Brain-Derived Neurotrophic Factor
BMI: Body Mass Index
C.R.: Congo red
DAB: Diaminobenzidine
DNP: Diabetic Neuropathic Pain
ELISA: Enzyme-Linked Immunosorbent Assay
FST: Forced Swim Test
GABA: Gamma-Aminobutyric Acid
GFAP: Glial Fibrillary Acidic Protein
GPx: Glutathione Peroxidase
GSH: Reduced Glutathione
H&E: Hematoxylin and Eosin
HDL-C: High-Density Lipoprotein Cholesterol
HFHS: High-Fat High-Sucrose diet
HFHSD: High-Fat High-Sucrose Diet
HFD: High-Fat Diet
HOMA-IR: Homeostatic Model Assessment for Insulin Resistance
hs-CRP: High-Sensitivity C-Reactive Protein
Inos: Inducible Nitric Oxide Synthase
IL-6: Interleukin-6
LDL-C: Low-Density Lipoprotein Cholesterol
MDA: Malondialdehyde
MDD: Major Depressive Disorder
NO: Nitric Oxide
PC: Protein Carbonyl
ROS: Reactive Oxygen Species
STZ: Streptozotocin
T.B.: Toluidine blue
TC: Total Cholesterol
TG: Triglycerides
TNF-α: Tumor Necrosis Factor-alpha
TST: Tail Suspension Test
T2DM: Type 2 Diabetes Mellitus
VLDL-C: Very Low-Density Lipoprotein Cholesterol
WD: Western Diet
